# Tactile cues are more intrinsically linked to motor timing than visual cues in visual-tactile sensorimotor synchronization

**DOI:** 10.3758/s13414-023-02828-9

**Published:** 2024-01-23

**Authors:** Michelle K. Huntley, An Nguyen, Matthew A. Albrecht, Welber Marinovic

**Affiliations:** 1 School of Population Health, Curtin University, Perth, Western Australia Australia; 2 School of Psychology and Public Health, La Trobe University, Wodonga, Victoria Australia; 3 Western Australia Centre for Road Safety Research, School of Psychological Science, University of Western Australia, Perth, Western Australia Australia

**Keywords:** Sensorimotor synchronization, Simultaneity judgment, Temporal binding window, Visual-tactile, Multisensory integration

## Abstract

Many tasks require precise synchronization with external sensory stimuli, such as driving a car. This study investigates whether combined visual-tactile information provides additional benefits to movement synchrony over separate visual and tactile stimuli and explores the relationship with the temporal binding window for multisensory integration. In Experiment 1, participants completed a sensorimotor synchronization task to examine movement variability and a simultaneity judgment task to measure the temporal binding window. Results showed similar synchronization variability between visual-tactile and tactile-only stimuli, but significantly lower than visual only. In Experiment 2, participants completed a visual-tactile sensorimotor synchronization task with cross-modal stimuli presented inside (stimulus onset asynchrony 80 ms) and outside (stimulus-onset asynchrony 400 ms) the temporal binding window to examine temporal accuracy of movement execution. Participants synchronized their movement with the first stimulus in the cross-modal pair, either the visual or tactile stimulus. Results showed significantly greater temporal accuracy when only one stimulus was presented inside the window and the second stimulus was outside the window than when both stimuli were presented inside the window, with movement execution being more accurate when attending to the tactile stimulus. Overall, these findings indicate there may be a modality-specific benefit to sensorimotor synchronization performance, such that tactile cues are weighted more strongly than visual information as tactile information is more intrinsically linked to motor timing than visual information. Further, our findings indicate that the visual-tactile temporal binding window is related to the temporal accuracy of movement execution.

**Supplementary Information:**

The online version contains supplementary material available at 10.3758/s13414-023-02828-9.

Navigating through our sensory-filled environment often involves little conscious effort. Yet what is occurring in our brains is a complex interplay between sensory and motor processes. This sensory and motor interplay occurs within a millisecond time range (Mauk & Buonomano, 2004), such that sensory information is integrated and actions are performed almost in synchrony (Mates et al., 1994; Roy et al., 2017). For sensory and motor processes to occur almost in synchrony, there is a reliance on temporal processing to facilitate the rapid integration of sensory information, and execute well-timed movements in response to external sensory cues (Mauk & Buonomano, 2004). The temporal nature of these sensorimotor processes has been investigated using sensorimotor synchronization tasks, which measure our ability to perform rhythmic actions in synchrony with external sensory stimuli (Iversen & Balasubramaniam, 2016; Mates et al., 1994).

Sensorimotor synchronization is an important skill that involves simultaneously anticipating the temporal pattern of external sensory information, integrating this sensory information, and performing coordinated actions in time with this information (Mates et al., 1994). For example, when driving a car, the driver monitors traffic conditions while performing coordinated actions with their arms and legs in response to changes in external sensory cues, such as when the traffic light changes color and the driver responds by accelerating, slowing down, or stopping the vehicle. Also, when walking with a friend, your walking pattern often falls into line with theirs, or when listening to music you tap your foot along to the beat of a song—these are all ways we intrinsically synchronize our actions to external sensory stimuli. In an experimental setting, sensorimotor synchronization is typically measured using tasks that require participants to press a button with their finger (i.e., finger-tap) in time with external sensory stimuli (Aschersleben, 2002).

Several models have been proposed to understand the processes involved in sensorimotor synchronization. The linear phase correction model highlights the importance of adjustments (“corrections”) made by the central nervous system (CNS) to the timing (“phase”) of movement execution (Schulze & Vorberg, 2002). These adjustments to the timing of movement execution reduce temporal discrepancies between the presentation of the sensory cue and movement execution, thereby improving the temporal accuracy of the response. In sensorimotor synchronization, the timing discrepancy between the presentation of the sensory cue and the response is known as the asynchrony. If the response (i.e., finger tap) occurs after the sensory stimulus, the asynchrony is positive, but it can be negative if the response precedes the external stimulus (Repp, 2005). Therefore, according to the linear phase correction model, the CNS corrects the phase of movement execution to reduce the time difference or asynchrony between stimulus and response in sensorimotor synchronization tasks. While the linear phase correction model explains the timing of our actions in relation to sensory cues, the maximum likelihood estimate (MLE) model explains how the reliability of the sensory cues themselves can affect integration.

In the MLE model, weights are assigned to each cross-modal cue, which results in a multisensory estimate that may be more precise than any unisensory estimate alone (Alais & Burr, 2004; Ernst & Banks, 2002). Therefore, this weighted multisensory estimate with higher precision may bias movement synchronization towards the cross-modal cue with the highest reliability. A highly reliable sensory cue would be consistent and accurate in predicting the appropriate timing for the motor response to occur, increasing the chances of success in a task. In the case of sensorimotor synchronization with cross-modal cues, movement synchrony would be more closely aligned with the sensory cue (or combined multisensory cue) that has the highest weight as this cue provides the most reliable sensory estimate of the appropriate time to act. As an extension of the MLE model, causal inference models are also relevant in the context of our study.

In addition to MLE models that focus on the weight of the reliability of sensory cues, the causal inference model proposes that the brain uses the current available multisensory information in combination with prior knowledge about the sensory stimuli to make predictions about the common cause of the cues (Körding et al., 2007; Parise et al., 2012). In the context of sensorimotor synchronization, information from previous trials would inform the individual about the timing of the multisensory cues and that the sensory stimuli originated from a common cause (Elliott et al., 2010, 2014). This prior knowledge would be weighted based on the result of the action. In other words, feedback about the success (or the lack of) would be important to form priors. This prior knowledge can then be used to reduce uncertainty associated with differences in the timing of sensory cues, improving the asynchrony between the multisensory cues and movement execution. Bayesian causal inference models offer an extension to the original causal inference model in that they consider the likelihood of the causal sources of multiple sensory cues given the prior knowledge and the available sensory information, and assign a probability to the likelihoods of the sources (Körding et al., 2007).

Sensorimotor synchronization with unisensory auditory stimuli has been well-established in different populations (Krause et al., 2010; Repp, 2005, 2010; Repp & Doggett, 2007; Repp & Su, 2013). Increasingly, research has explored the effect of unisensory auditory, visual, and tactile stimuli on sensorimotor synchronization and multisensory cross-modal combinations of these stimuli (Armstrong & Issartel, 2014; Elliott et al., 2010; Jin et al., 2019; Roy et al., 2017; Wing et al., 2010). As our environment contains an abundance of sensory information, using multisensory stimuli in experimental protocols allows for a more realistic and in-depth understanding about the influence of sensory information on the accuracy of movement execution (Shams & Seitz, 2008). Previous research shows that participants are typically less variable when synchronizing their actions with multisensory stimuli including audio-visual, audio-tactile and visual-tactile modalities, than respective unisensory stimuli alone (i.e., auditory, visual, or tactile stimuli; Armstrong & Issartel, 2014; Elliott et al., 2010; Jin et al., 2019; Roy et al., 2017; Wing et al., 2010). However, this multisensory benefit may be dependent upon sensory modality. For example, when synchronizing movement with simultaneously presented audio-tactile stimuli, sensorimotor synchronization variability was similar between multisensory stimuli and auditory alone, with variability only increasing marginally with tactile-alone stimuli (Elliott et al., 2010; Roy et al., 2017). Similarly, when synchronizing movement with simultaneously presented visual-tactile stimuli there was no difference in variability between multisensory stimuli and tactile alone, with variability only increasing with visual-alone stimuli (Elliott et al., 2010). Although this reduction in sensorimotor synchronization variability with multisensory stimuli, compared with unisensory stimuli, may be small and dependent on sensory modality, it is still a consistent finding and therefore indicates that multisensory information improves the temporal precision of motor execution. However, to our knowledge, the only study to examine visual-tactile sensorimotor synchronization used a relatively small sample (*n* = 6; Elliott et al., 2010). Hence, further investigation is required to characterize sensorimotor synchronization with visual-tactile stimuli. Due to the crucial role visual-tactile information plays in motor control and the limited research on visual-tactile sensorimotor synchronization, the aim of Experiment 1 in the current study is to characterize sensorimotor synchronization with visual-tactile stimuli.

Further evidence for a unique multisensory effect on sensorimotor synchronization performance can be gleaned from studies that apply temporal jitter to one stimulus in a cross-modal pair. Typically, when temporal jitter is applied to one stimulus in a cross-modal pair, variability of sensorimotor synchronization increases; this effect is consistent across different cross-modal pairs of stimuli (audio-tactile, audio-visual, visual-tactile) and a range of temporal jitter from 20 ms to 160 ms (Elliott et al., 2010; Roy et al., 2017; Wing et al., 2010). As temporal jitter increases, the variability of sensorimotor synchronization also increases (i.e., variability was higher when temporal offsets were longer; Elliott et al., 2010; Roy et al., 2017; Wing et al., 2010). These results indicate that presenting cross-modal stimuli nonsimultaneously influences variability of movement execution in sensorimotor synchronization tasks. A potential explanation for this increase in variability in sensorimotor synchronization is the length of an individual’s temporal binding window (TBW) for multisensory integration. The TBW is the time range in which sensory information from multiple modalities is integrated into a single concept, which is then attributed to a concurrent perceptual event (Ernst & Bülthoff, 2004; Stein & Stanford, 2008; Wallace & Stevenson, 2014; Wallace et al., 2020). We can think of the TBW in terms of the causal inference model such that when multisensory stimuli are received within the TBW, they are attributed to the same cause. Since sensory information within the window is perceived as occurring simultaneously yet is received at different times across the brain, movement execution may be phase shifted towards the presentation of the later stimulus that is still within the TBW. The degree to which the movement is temporally shifted towards to later stimulus is likely dependent upon the relative reliability weighting between sensory cues. Hence, sensorimotor synchronization variability may increase (or decrease) depending on the modality of the stimulus that is temporally jittered within the TBW, and the weighting assigned to the sensory modality.

Despite the suggestion in previous literature that the TBW influences sensorimotor synchronization performance (Elliott et al., 2010; Repp, 2005; Roy et al., 2017), only one study to date has examined sensorimotor synchronization performance when stimuli are presented inside and outside the TBW (Elliott et al., 2014). Further, no studies have used the simultaneity judgment task to measure the TBW to establish a relationship between the window and sensorimotor synchronization performance. It is relevant to use the simultaneity judgment task as it is an established method for measuring the TBW (Chen et al., 2018; Hillock et al., 2011; Hillock-Dunn & Wallace, 2012; Powers et al., 2009; Stevenson et al., 2012a, b, 2013; Stevenson & Wallace, 2013). Therefore, the aim of Experiment 2 in the current study is to (1) investigate whether sensorimotor synchronization performance is differentially influenced by stimuli presented either inside or outside of the TBW, and (2) examine the relationship between sensorimotor synchronization performance and the length of the TBW.

In Experiment 1, our first hypothesis was that sensorimotor synchronization variability would be lower with cross-modal visual-tactile stimuli than with respective unimodal stimuli. We analyzed standard deviation to examine variability as we were interested in determining whether individuals showed enhanced precision in their tapping ability when synchronizing their tap with either (a) unimodal or bimodal stimuli and (b) visual or tactile sensory stimuli. Our second hypothesis was that higher sensorimotor synchronization variability would be related to a longer TBW. In Experiment 2, our first hypothesis was that sensorimotor synchronization temporal error would be higher when cross-modal stimuli were presented inside the TBW with a stimulus onset asynchrony (SOA) of 80 ms, than when one stimulus in the cross-modal pair was presented inside the TBW and the second outside the TBW, with an SOA of 400 ms. We examined mean asynchrony to investigate whether there were differences in sensorimotor synchronization accuracy when cross-modal sensory stimuli were presented both inside the TBW and when the second stimulus in the cross-modal pair was presented outside of the TBW, and whether the sensory modality influenced the accuracy of sensorimotor synchronization ability. Our second hypothesis was that sensorimotor synchronization temporal error would be lower when TBWs were narrower, compared with when TBWs were wider. In both experiments, we expect that the size of the TBW will be related to sensorimotor synchronization performance. A smaller TBW indicates multisensory stimuli are bound efficiently and are more likely to represent a “true” perception about events in the environment, whereas a larger (wider) TBW indicates multisensory stimuli are bound over a longer period. When stimuli are bound together over a longer period, there is more opportunity for irrelevant information to be bound with relevant information, which likely distorts the “true” cause of the stimuli and potentially inaccurately representing perceptual events in the environment (Wallace & Stevenson, 2014). Without an accurate perception of events in the environment it would be difficult to accurately synchronize movement with external sensory stimuli (Iarocci & McDonald, 2006).

## Experiment 1

### Method

#### Participants

Thirty-one participants were recruited for the study from an undergraduate University population. All participants recruited for the study received points towards their course as compensation for participating in the study. For Task 1 (sensorimotor synchronization task), all 31 participants were included in the data analysis (*M* = 20.77, *SD* = 2.31, range: 18–28 years old, 20 female). In Task 2 (simultaneity judgment task), seven participants were excluded from the analysis due to poor fit of their data. From visual inspection of the data, these participants were unable to discriminate between simultaneous and nonsimultaneous stimuli across the range of SOAs, which meant that we were unable to attain a reliable estimate of their TBW. Therefore, 24 participants were included in the data analysis (*M* = 20.83, *SD* = 2.50, 14 female). Our sample size was guided by previous literature (Armstrong & Issartel, 2014; Elliott et al., 2010, 2014; Lagarde & Kelso, 2006; Wing et al., 2010) and exceeds those commonly seen in similar studies in this area of research. All participants were right-hand dominant and free from any neurological conditions. The study was approved by Curtin University Human Research Ethics Committee. Experiments in this study were conducted in accordance with the Declaration of Helsinki. All participants gave written informed consent prior to testing and completed a demographics questionnaire.

#### Stimuli

Instructions for the tasks were displayed on a 19-in. Dell LCD computer monitor (60-Hz refresh rate). The experiment was programmed in MATLAB (Version 2015b), and the instructions and stimulus triggers were programmed using Psychtoolbox (Version 3.0.8). The visual stimuli were two 5-mm green light emitting diodes (LEDs; 10,000 mcd) inside two frosted Perspex blocks fixed to a black stand placed in the centre of the computer monitor. The tactile stimuli were two 10 × 3.4-mm shaftless vibration motors (Pololu Corporation, Las Vegas, NV; Pololu item #1636) attached to the index finger and middle finger on the left hand (nondominant) with elastic fabric bands. In Task 1, the LED on the right side of the board and the vibration motor on the left index finger was used, and each stimulus were presented for 64 ms. In the Task 2, left and right LEDs were presented, and the second vibration motor attached to the left middle finger was used, and each stimulus was presented for 50 ms. Participants completed all tasks in a dimly lit room. In both tasks, participants were approximately 60 cm from the visual stimuli, which were in front of the computer monitor and in central view. For the tactile stimuli, participants were instructed to place their hand on the desk in front of them and were allowed to place the hand in a comfortable position.

#### Experimental procedures

##### Task 1: Sensorimotor synchronization

**Fig. 1:**
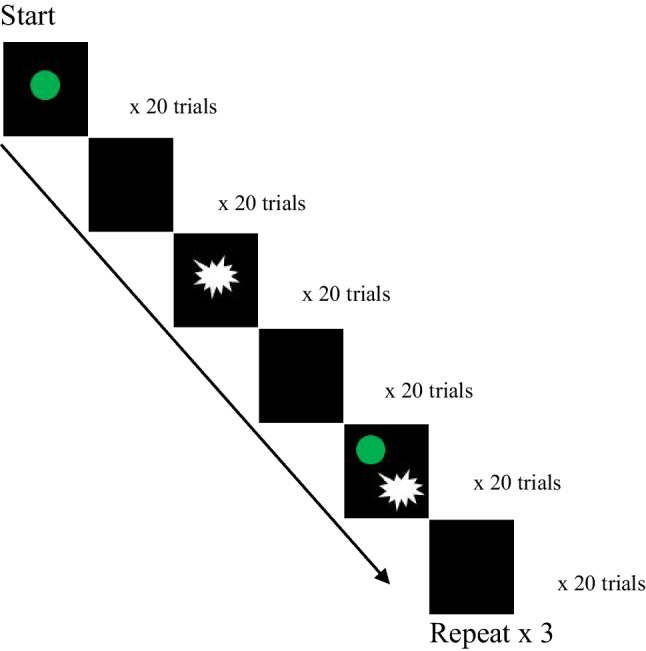
Graphical representation of the trial design for Task 1: Sensorimotor synchronization. Green circles represent the green LED (visual stimulus), the white star represents the vibration motor (tactile stimulus), and the black square represents the blank screen presented during wash-out trials. Trials were presented at either 600 ms ISI or 1200 ms ISI; the 600 ms and 1200 ms ISIs were presented in separate blocks, counterbalanced between participants. Pictured here is Combination 1 (of six possible combinations that were pseudorandomized between participants) which follows: 20 visual trials, 20 wash-out trials, 20 tactile trials, 20 wash-out trials, 20 visual-tactile trials, 20 wash-out trials, repeated 3 times. The remaining combinations (not pictured): were (2) visual, wash-out, visual-tactile, wash-out, tactile, wash-out, repeat × 3; (3) tactile, wash-out, visual, wash-out, visual-tactile, wash-out, repeat × 3; (4) tactile, wash-out, visual, wash-out, visual-tactile, wash-out, repeat × 3; (5) visual-tactile, wash-out, tactile, wash-out, visual, wash-out, repeat × 3; (6) visual-tactile, wash-out, visual, wash-out, tactile, wash-out, repeat × 3. (Color figure online)

Participants were instructed to keep their right index finger resting on the right arrow key on a computer keyboard and tap their finger in synchrony with the sensory stimuli: either a visual stimulus, tactile stimulus, or a visual-tactile stimulus, and to continue tapping at the same pace when the stimuli disappeared. We used a computer keyboard to record the timing of the finger tapping, as previous research has shown that using PsychToolbox in MATLAB accurately records the timing of responses from key presses (Navracsics & Darzhinova, 2020), and that keyboards only introduce minor jitter in the millisecond range, therefore recording reaction time with good accuracy (Anwyl-Irvine et al., 2021; De Clercq et al., 2003). The sensorimotor synchronization task consisted of 480 trials for each ISI (total of 960 trials). For both the 600 ms ISI and 1200 ms ISI, there were 480 trials; 240 sensory trials (80 visual, 80 tactile, 80 visuo-tactile) and 240 nonsensory trials in which no sensory stimuli were presented. As shown in Fig. 1, the sensory and nonsensory trials were presented in one of six possible combinations. Within each combination, 20 sensory trials (either visual, tactile, or visuo-tactile) were followed by 20 nonsensory trials, and this pattern was repeated three times. The combination order of the sensory condition and the ISI was pseudorandomized between participants to reduce potential order effects. In the nonsensory trials, the sensory stimuli were suppressed so that the computer monitor only displayed a black screen and no tactile or visual stimuli was presented. We opted to suppress the sensory stimuli, rather than simply presenting a black screen, to maintain the exact timing of the sensory trials. These nonsensory trials were used as a wash-out period between sensory trials in a different modality to avoid any carry over effects associated with the timing of stimuli in any given modality. Participants were instructed to continue tapping during the wash-out period to maintain the rhythmic pattern of the stimuli for when the sensory trials next commenced. There were two blocks of trials at different interstimulus intervals (ISIs)—600 ms and 1200 ms—to observe any differences in sensorimotor synchronization variability between sub- and suprasecond timing intervals. Participants wore industrial passive headphones to prevent the sound of the key press being used as feedback about their timing. Practice blocks were completed prior to each experimental block and were in the same combination order as the experimental blocks. Each practice block consisted of 60 sensory and 60 “wash-out” (nonsensory) trials.

##### Task 2: Simultaneity judgment task

**Fig. 2:**
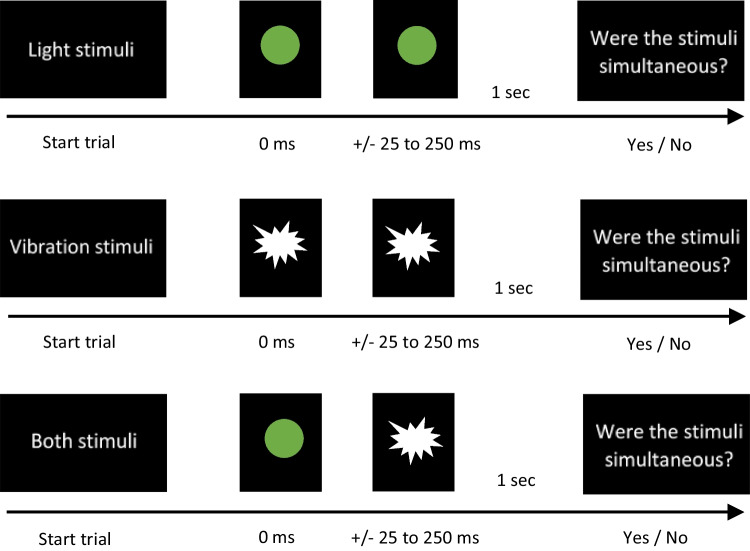
Graphical representation of the trials for visual (“light”), tactile (“vibration”), and visual-tactile (“both”) stimuli presented in the simultaneity judgment task. Stimuli were presented at these SOAs: ±0, 25, 50, 75, 100, 125, 150, 175, 200, 225, 250 ms

Participants completed a simultaneity judgment task with visual and tactile stimuli. The beginning of each trial started with the sensory type written on the screen (e.g., visual stimuli, tactile stimuli, or both stimuli; see Fig. 2). In the visual stimuli condition, both (left and right) LEDs were presented in each trial; in the tactile stimuli condition, both (index and middle finger) vibration motors were presented in each trial; and in the both stimuli condition, one LED and one vibration motor were presented (right LED and index finger) in each trial. The two sensory stimuli were presented either together (i.e., simultaneously) or at varying SOAs from ±25 ms to 250 ms, increasing incrementally in 25-ms steps. When stimuli were presented at negative SOAs, it means one stimulus preceded the other (e.g., when SOAs were negative the visual stimulus preceded the tactile stimulus, and when SOAs were positive, the tactile stimulus preceded the visual stimulus). Following the presentation of either visual stimuli, tactile stimuli, or both stimuli (cross-modal stimuli), the question “Were the stimuli simultaneous?” appeared on the screen, and participants responded “yes” by clicking the left mouse button or “no” by clicking the right mouse button. Participants were instructed to respond as quickly as possible following the second stimulus. There were 10 trials for each condition (±SOA × sensory pair), totaling 630 trials. Participants completed 12 practice trials in pseudorandomized order before commencing the experiment. The order of the sensorimotor synchronization task and simultaneity judgment task were counterbalanced between participants.

#### Data analysis

In Task 1, the time between stimulus presentation and the finger-tap is referred to as the asynchrony, and the standard deviation of the asynchrony is used as the measure of sensorimotor synchronization ability (Repp, 2005; Repp & Su, 2013). Asynchrony standard deviation was calculated across all trials for each participant. As such, the asynchrony standard deviation (*SD*) was included in the statistical analysis for Task 1, in which linear mixed models (LMM) were conducted using the gamlj mixed function in RStudio (gamlj package Version 2.6.5, Gallucci, 2019; RStudio, Version 3.5.2, RStudioTeam, 2015) to test main effects and interactions. Post-hoc tests were conducted using the estimated marginal means (part of the gamlj package in RStudio), with the Holm method used to correct for multiple comparisons (Holm, 1979), and Satterthwaite method to correct the degrees of freedom. In Task 1, the effect of stimulus type (visual, tactile, visual-tactile) and time (ISI; 600 ms, 1200 ms) on sensorimotor synchronization was analyzed using LMMs, with stimulus type and time as the fixed factors and participants as the random factor.

In Task 2, the percentage of simultaneous responses (i.e., responding “yes” to “Were the stimuli simultaneous?” after each trial) were averaged for each participant at each SOA and fitted with a model-free line was fitted to each participants data across SOAs using the modelfree package in R Studio (Model-Free Estimation of a Psychometric Function, Version 1.2; Zychaluk & Foster, 2009). The modelfree function is a parameter free method that has no assumptions about the shape of the data. This function provides an alternative to commonly used methods, such as Gaussian or sigmoid functions, which are commonly used to fit data for the perception of simultaneity (Costantini et al., 2016; Hillock et al., 2011; Hillock‐Dunn & Wallace, 2012; Migliorati et al., 2020; Moro & Steeves, 2018; Noel et al., 2016, 2017; Powers et al., 2009; Stevenson et al., 2014a, 2018; Stevenson & Wallace, 2013; Venskus et al., 2021). For each participant, we estimated the width of the TBW by identifying the half-way point between 0% and the maximal peak of simultaneity perception on the *y*-axis, and where this half-way point intersects the model-free fit on the *x*-axis. Only visual leading visual-tactile trials have been included in the calculation of the TBW and statistical analysis as when the data was inspected for the tactile leading visual-tactile trials it was found that a number of participants had the same width window as the longest SOA, thus indicating that the SOAs for Experiments 1 and 2 in this study were not long enough to measure the width of the TBW. Spearman’s rank-order correlation coefficient was calculated to examine the relationship between the visual leading visual-tactile TBW and visual-tactile sensorimotor synchronization ability in Task 1.

### Results

#### Task 1: Sensorimotor synchronization

**Fig. 3:**
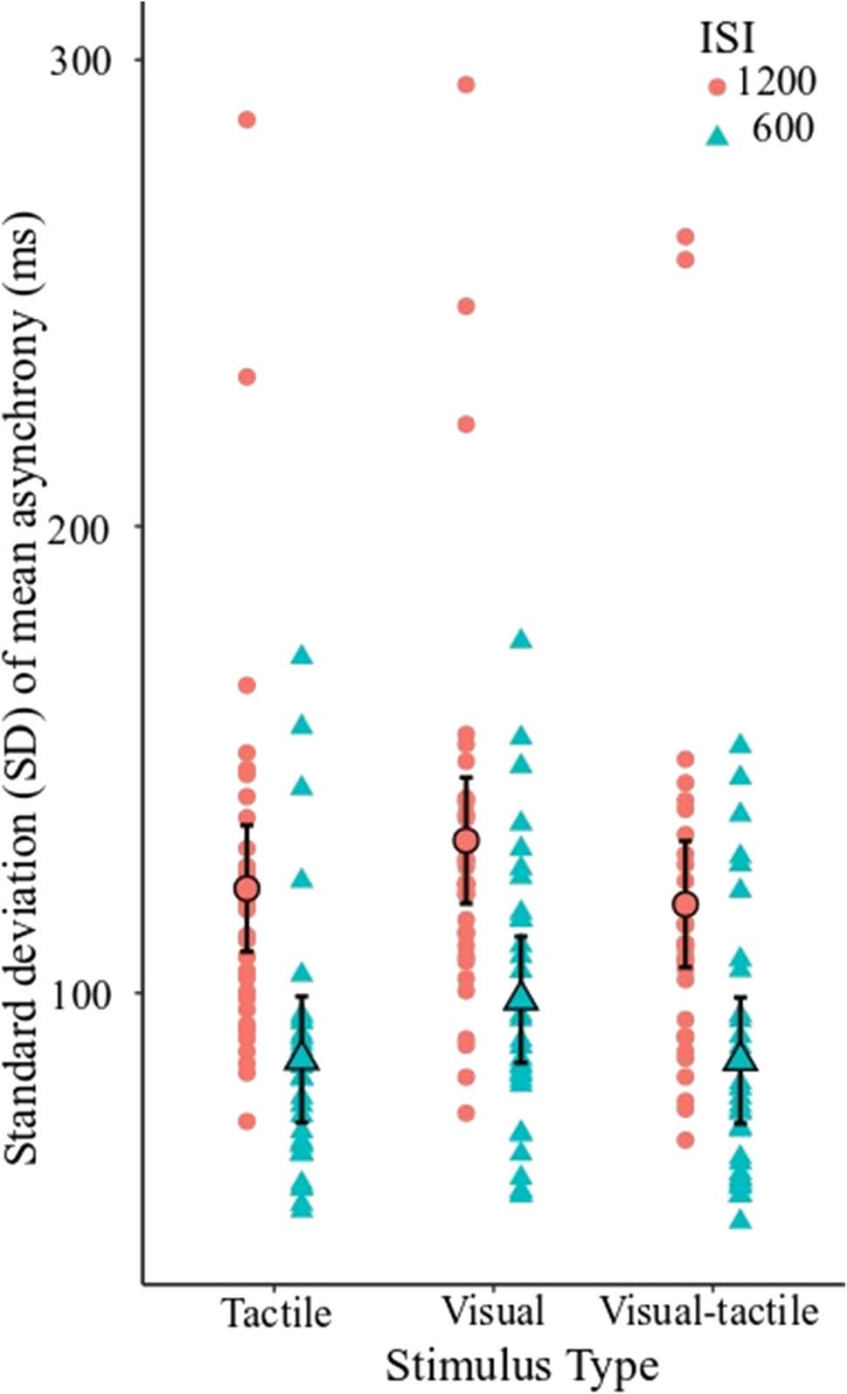
Sensorimotor synchronization asynchrony standard deviation for visual, tactile and visual-tactile conditions. Red circles represent the 1200-ms ISI condition, and the blue triangles represent the 600-ms ISI condition. Group-level data are shown in black outline, and subject-level data are shown without outline. Error bars represent 95% confidence intervals. (Color figure online)

For asynchrony variability, results from the LMM showed main effects of stimulus type, *F*(2, 150) = 6.37, *p* = 0.002, and time, *F*(1, 150) = 109.97,* p* < 0.001, but no Stimulus Type × Time interaction, *F*(2, 150) = 0.086, *p* = 0.92 (see Table 1 for group mean asynchrony and standard deviation). Post hoc tests indicate that sensorimotor synchronization ability was more accurate when tapping in synchrony with the visual-tactile stimuli than visual alone (*p* = 0.003), but there was no difference in sensorimotor synchronization ability when tapping in synchrony with visual-tactile stimuli and tactile alone (*p* = 0.661). When comparing differences between unimodal conditions, sensorimotor synchronization ability was more accurate when tapping in synchrony with tactile stimuli alone than visual alone (*p* = 0.010; see Fig. 3). For all sensory modality conditions, the finger-tap movement preceded the sensory stimuli, consistent with previous literature (Aschersleben, 2002). Overall, mean asynchrony was lower for the 1200-ms condition than for the 600-ms condition (see Supplementary Material for LMM with mean asynchrony).

**Table 1:** Group mean asynchrony and standard deviation for 600 ms and 1,200 ms ISI conditions

	600 ms		1,200 ms	
	*M*	*SD*	*M*	*SD*
Visual	−71.46	38.23	−56.45	46.14
Tactile	−64.99	39.04	−55.65	43.05
Visual-tactile	−68.01	36.63	−57.32	35.15

#### Task 2: Simultaneity judgment task (*N* = 24)

**Fig. 4:**
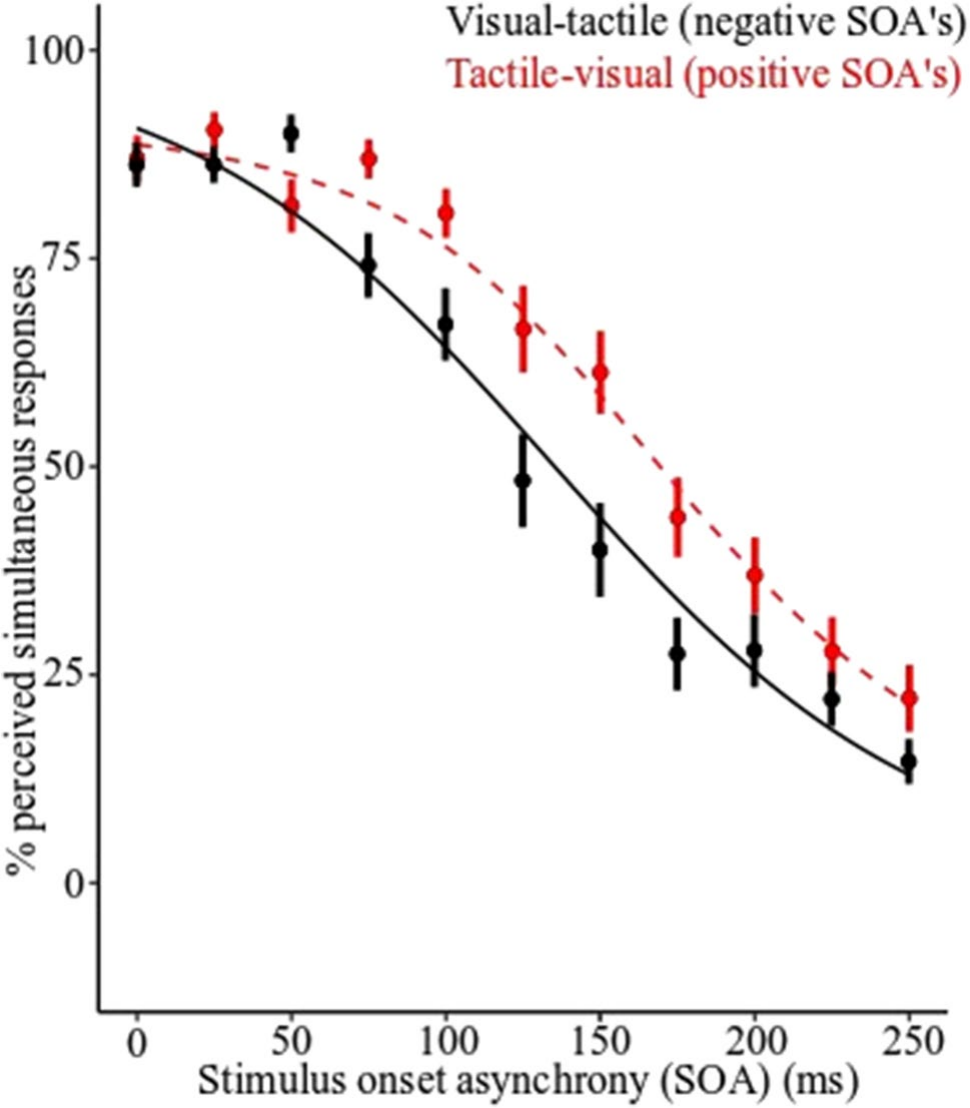
Group percentage of perceived simultaneity for visual leading visual-tactile stimuli (VT) and tactile leading visual-tactile stimuli (TV). Error bars represent the standard error of the model estimates

Results showed that mean width of the visual leading visual-tactile TBW was 149 ms (VT-TBW; *SD* = 55 ms) and the mean width of the tactile-leading visual-tactile TBW was 180 ms (TV-TBW; *SD* = 48 ms; see Fig. 4).

**Fig. 5:**
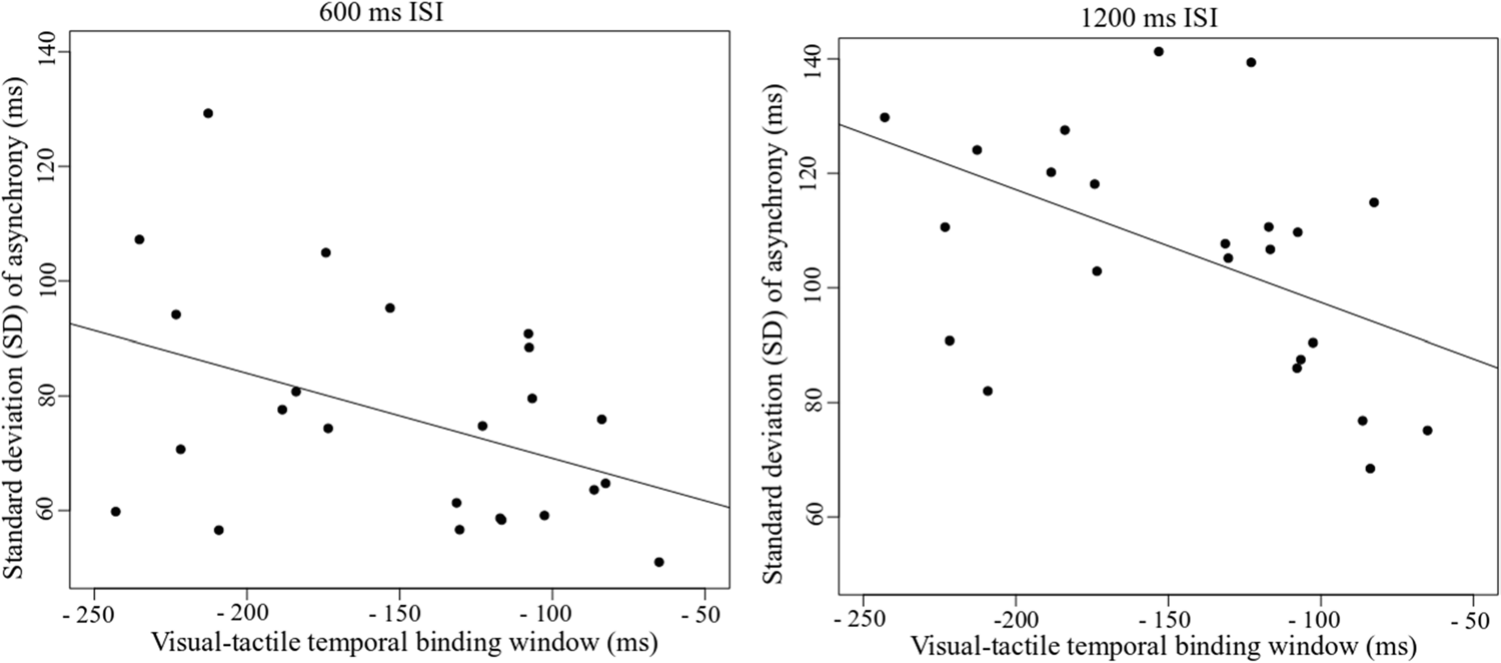
Scatterplots showing correlations between sensorimotor synchronization ability (standard deviation asynchrony) at 600 ms and the visual leading visual-tactile TBW (top), and between sensorimotor synchronization ability at 1200 ms and the visual-tactile TBW (bottom)

There was a significant correlation between the visual leading visual-tactile TBW and sensorimotor synchronization asynchrony standard deviation (as a measure of sensorimotor synchronization ability) in the 1200-ms ISI condition (*r* = −0.56, *p* ≤ 0.001), but no correlation in the 600-ms ISI condition (*r* = −0.34, *p* = 0.100). Despite there being no correlation in the 600-ms ISI condition, Fig. 5 shows the pattern of results is similar between the 600-ms and 1200-ms conditions. Taken together, these results indicate that greater variability in sensorimotor synchronization ability—indicating poorer movement synchrony—may rely on the engagement of similar mechanisms that are engaged when temporally binding sensory information within a specified time (i.e., the TBW).

## Experiment 2

### Method

#### Participants

Thirty-seven participants were recruited for the study from the same undergraduate university population as in Experiment 1. As with Experiment 1, all participants recruited for the study received points towards their course as compensation for participating in the study. In Task 1 (sensorimotor synchronization with stimuli inside and outside the window), nine participants were excluded from the data analysis: eight due to technical issues and one due to an incomplete data set. Therefore, a total of 28 participants were included in the data analysis for Task 1 (*M* = 21.18, *SD* = 3.15, range: 18–32 years old, 20 female). In Task 2 (simultaneity judgment task), four participants were excluded: three due to a poor fit of their data (as outlined in Experiment 1, Task 2) and one due to an incomplete data set. Therefore, a total of 33 participants were included in the data analysis for Task 2 (*M* = 21.30, *SD* = 3.04, range: 18–32 years old, 22 female). All participants reported normal-to-corrected vision and were free from any neurological conditions. The study was approved by Curtin University Human Research Ethics Committee. All participants gave written informed consent prior to testing and completed a demographics questionnaire.

#### Stimuli

The same stimuli as Experiment 1 were used - the visual stimulus was a green LED and the tactile stimulus was a shaftless vibration motor. The LED and the vibration motor were attached to the top third part of the left index finger with an elastic fabric band and secured with micropore tape. The stimuli were in this position for both experimental tasks.

#### Experimental procedures

##### Task 1: Sensorimotor synchronization with stimuli inside and outside window

Participants completed the sensorimotor synchronization task with the same visual and tactile stimuli as in Experiment 1. There were two blocks of trials—one block of trials consisted of visual-only and visual-tactile trials, the other block of trials consisted of tactile-only and tactile-visual trials. The order of block presentation was counterbalanced across participants. At the beginning of each block of trials, participants were instructed to press the right arrow key on the computer keyboard in synchrony with the visual stimulus for the visual-only and visual-tactile trials, or the tactile stimulus for the tactile-only and tactile-visual trials. Within each bimodal condition—that is, visual-tactile and tactile-visual—the second stimulus in the pair was presented with a SOA of 80 ms or 400 ms. The 80-ms SOA meant that both stimuli were presented inside the TBW, and the 400-ms SOA meant that the first stimulus was presented inside the TBW and the second stimulus was presented outside the TBW. Prior to determining the inside and outside TBW SOAs (80 and 400 ms, respectively), we had previously conducted the visual-tactile simultaneity judgment task in two experiments with different groups. In these studies, the mean width of the visual-tactile window was 149 ms (Experiment 1, Task 2 in the current manuscript) and 123 ms (Huntley et al., 2023) and the shortest width was ~80 ms. From this data we estimated that 80 ms would be inside the TBW, and 400 ms would be well outside the window. We have referred to the first stimulus in the pair as the “attended” to stimulus as the participants were instructed to tap in synchrony with this stimulus, and the second stimulus in the pair as the “irrelevant” stimuli. Note however that we did not specifically manipulate attention as we did not explicitly instruct participants to maintain attention on the first stimulus and ignore the second stimulus. The “attended” to stimulus was presented at the same time in both unimodal and cross-modal conditions and only the timing of the “irrelevant” stimulus varied as it was presented either “inside” (SOA 80 ms) or “outside” (SOA 400 ms) the TBW. Each block consisted of 60 unimodal trials (visual or tactile), followed by 60 cross-modal trials. The ISI for unimodal and cross-modal blocks was 1200 ms. Therefore, there were six conditions: visual only, tactile only visual-tactile attend (in), visual-tactile attend (out), tactile-visual attend (in) and tactile-visual attend (out). Practice blocks were completed before each experimental block, and experimental blocks were counterbalanced across participants.

##### Task 2: Simultaneity judgment task

Participants completed a simultaneity judgment task with cross-modal visual-tactile stimuli only. The visual and tactile stimuli used were the same as those in the “both stimuli” condition in Experiment 1, Task 2. Consistent with Experiment 1, when SOAs were negative the visual stimulus preceded the tactile stimulus, and when SOAs were positive the tactile stimulus preceded the visual stimulus. Positive SOAs ranged from 25 to 225 ms, and negative SOAs ranged from −25 to −250 ms, and both increased in 25 ms increments. All instructions to participants were consistent with Experiment 1, Task 2. There were 10 trials for the 0 ms condition and each SOA, totaling 200 trials. Participants completed practice trials in pseudorandomized order before commencing the experiment. The order of the sensorimotor synchronization task and simultaneity judgment task were counterbalanced between participants.

#### Data analysis

In Task 1, the mean asynchrony of the unimodal trials was used as the baseline condition and was subtracted from the respective mean asynchrony of the cross-modal condition (e.g., visual attend/tactile irrelevant (in) mean asynchrony minus visual baseline mean asynchrony) to create an asynchrony difference score for each condition. Therefore, the difference score represents the effect of the combined cross-modal stimuli after removing the modality-specific effects on mean asynchrony. The effect of attended sensory modality (visual attend/tactile irrelevant, tactile attend/visual irrelevant) and the timing of the second stimulus (80 ms, 400 ms) on sensorimotor synchronization was analyzed using LMM, with sensory modality and time as the fixed factors and participants as the random factor. LMM’s were conducted to test main effects and interactions, and post hoc tests were conducted for further analysis. The statistical software and packages used were consistent with Experiment 1.

In Task 2, the TBW was calculated for each participant using the same method as Experiment 1. Spearman’s rank-order correlation coefficient was calculated to examine the relationship between the visual leading visual-tactile TBW and the mean asynchrony difference score on the sensorimotor synchronization task. Due to exclusions from Task 1 and Task 2, twenty-six participants were included in the correlations between sensorimotor synchronization mean asynchrony and the TBW.

### Results

#### Task 1: Sensorimotor synchronization with stimuli inside and outside window

**Fig. 6:**
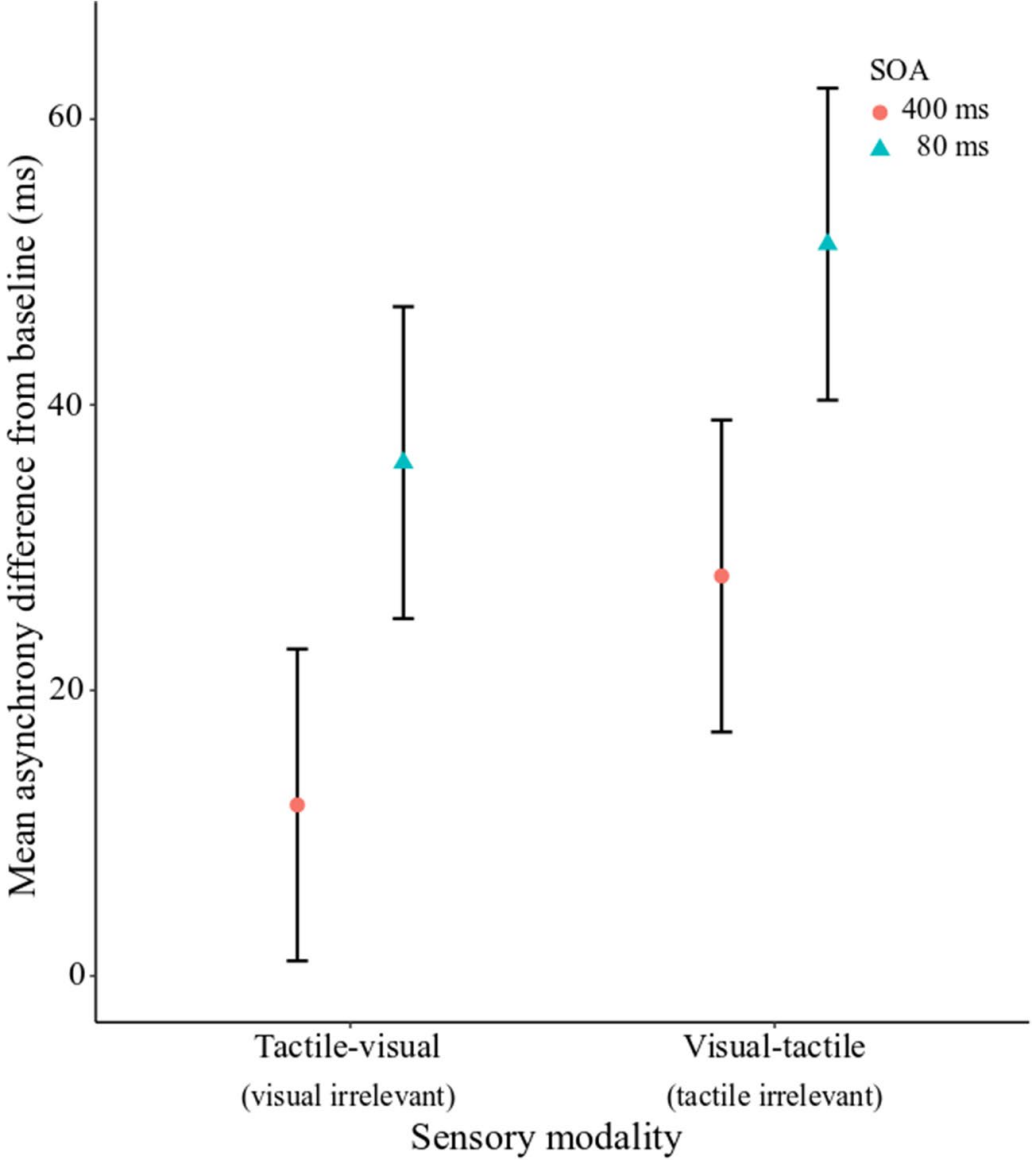
Sensorimotor synchronization mean asynchrony difference score (cross-modal minus unimodal per condition) for tactile-visual (tactile attend/visual irrelevant) and visual-tactile (visual attend/tactile irrelevant) for SOAs 80 ms and 400 ms. Error bars represent the standard error of the model estimates

Temporal error during the sensorimotor synchronization task was measured using the mean asynchrony across trials for each condition. The mean asynchrony for each condition was assessed using a LMM with fixed effects of Time (80 ms, 400 ms) and Sensory Modality (visual-tactile/tactile irrelevant, tactile-visual/visual irrelevant), and a Time × Sensory Modality interaction. Results from the LMM showed a main effect of Time, *F*(1, 81) = 4.89, *p* = 0.029, no effect of Sensory Modality, *F*(1, 81) = 2.16, *p* = 0.145, and no Time × Sensory Modality interaction, *F*(1, 81) = 0.001, *p* = 0.923. As shown in Fig. 6, temporal error (mean asynchrony difference from baseline) was lower for the tactile attend (visual irrelevant) than the visual attend (tactile irrelevant) for stimuli presented both inside and outside the TBW although this effect was not significant. See Supplementary Material for the LMM with sensorimotor synchronization variability (standard deviation difference from baseline) and stimulus modality.

#### Task 2: Simultaneity judgment task

**Fig. 7:**
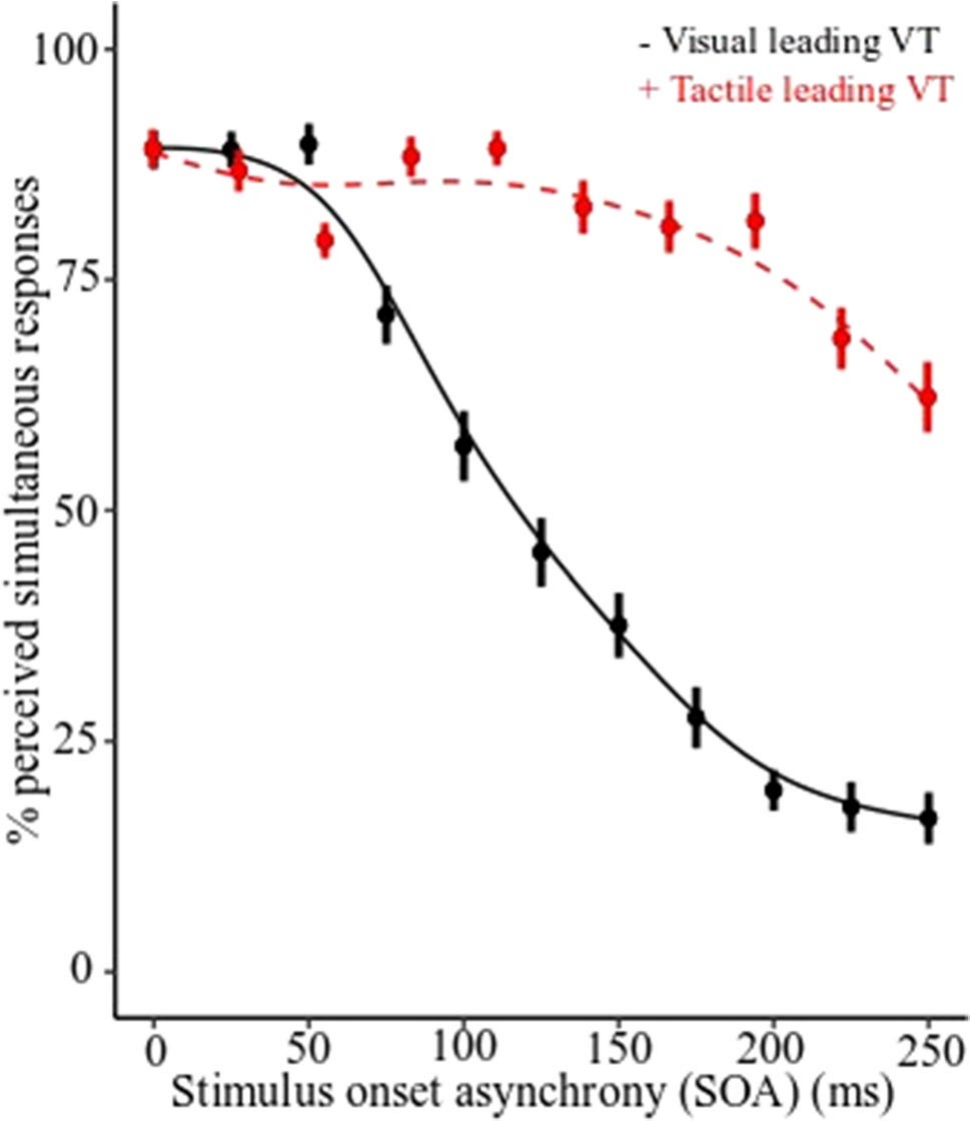
Group percentage of perceived simultaneity for visual leading visual-tactile stimuli (VT) and tactile leading visual-tactile stimuli (TV). Error bars represent the standard error of the model estimates

Results from the simultaneity judgment task showed that the mean width of the VT-TBW for the group was 139 ms (*SD* = 50 ms) and the mean width of the TV-TBW was 179 ms (*SD* = 87 ms; see Fig. 7). Caution should be exercised when interpreting the mean width of the TV-TBW in Experiment 2 as a subgroup of participants possessed a window equal to the largest SOA, which indicates that their TV-TBW is likely to be longer than we were able measure with the SOAs included in the task. Another subgroup of participants had a TV-TBW equal to 0 ms, which indicates that they were unable to distinguish between simultaneous and non-simultaneous stimuli therefore a model-free curve could not be fitted to the data.

**Fig. 8:**
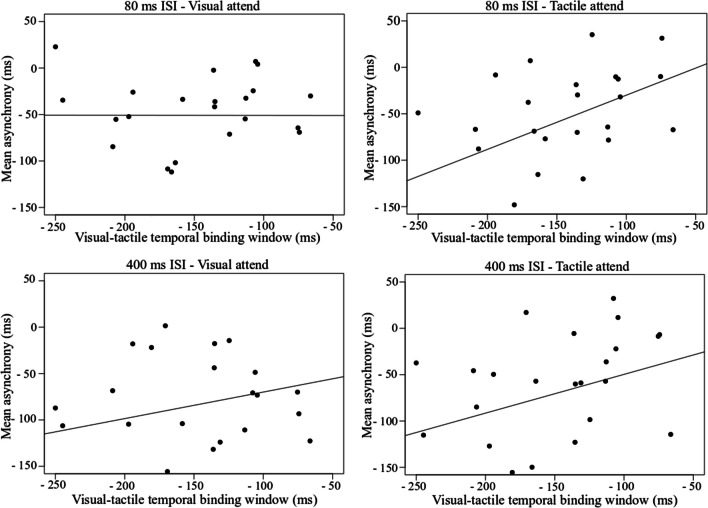
Top row scatterplots show correlations between sensorimotor synchronization mean asynchrony at 80-ms ISI (i.e., when both stimuli are presented within the TBW) and the visual-tactile (visual leading TBW) when participants attend to the visual and tactile stimuli. Bottom row scatterplots show correlations between sensorimotor synchronization mean asynchrony at 400-ms ISI (i.e., when one stimulus is presented within the TBW and the second stimulus is presented outside the TBW) and the visual-tactile (visual leading) TBW when participants attend to the visual and tactile stimuli

For the tactile attend condition, there was a significant correlation between the TBW and sensorimotor synchronization mean asynchrony when both stimuli were presented inside the TBW (80 ms), (*r* = −0.42, *p* = 0.04), but no correlation when the second stimulus was presented outside the TBW (400 ms), (*r* = 0.36, *p* = 0.07). Although, not statistically significant, the pattern of results for tactile attend (out) was similar to tactile attend (in), suggesting that tactile information is relied on more than visual information in both tasks (see Scatterplots, Fig. 8). The results can be interpreted as evidence that information bound within the TBW likely influences the timing accuracy of movement execution. In the visual attend condition, there was no significant correlation between the TBW and sensorimotor synchronization mean asynchrony inside, (*r* = 0.1, *p* = 0.65), or outside the window (*r =* 0.12, *p* = 0.56).

### Discussion

The aim of Experiment 1 was to characterize sensorimotor synchronization with visual-tactile stimuli by comparing variability in movement synchronization between cross-modal and unimodal stimuli. Although we found that sensorimotor synchronization with visual-tactile stimuli was less variable than with visual stimuli alone, there was no difference between visual-tactile stimuli and tactile stimuli alone. Therefore, our hypothesis about multisensory stimuli providing a benefit to performance in sensorimotor synchronization was only partially supported. These results are reasonably consistent with previous literature, which found that there was no difference in sensorimotor synchronization variability between audio-tactile stimuli and auditory alone, but there was a difference between audio-tactile stimuli and tactile alone (Roy et al., 2017). Similarly, with visual-tactile stimuli, sensorimotor synchronization variability was comparable between visual-tactile stimuli and tactile alone, but there was a noticeable difference in variability between visual-tactile stimuli and visual alone (Elliott et al., 2010, Fig 2C). Previous research has shown differential sensorimotor synchronization effects across sensory modality. In an audio-tactile bimanual coordination task, a finger flexion was more tightly coupled with sound and finger extension with touch (Lagarde & Kelso, 2006). Our results show that when the sensory cue (tactile) is in the same modality as the action (finger-tapping) our ability to synchronize our movements with sensory information is enhanced. Supporting this idea, we have shown that the TBW for tactile-tactile stimuli is very narrow indicating that tactile information is integrated rapidly (Huntley et al., 2023), which enables faster execution of an action.

Our results are consistent with the linear phase correction and MLE models as sensorimotor synchronization to sensory cues is related to the task-type in addition to the stability of the sensory cue itself. Our results from Experiment 1, Task 1 (SMS) indicate that tactile cues are assigned a higher weight by the CNS, which increases the reliance on tactile cues for temporal regularity of incoming sensory information. As the tactile cue is more intrinsically related to motor timing than the visual cue, the CNS phase shifts the execution of the finger tap to be temporally aligned with the presentation of the tactile cue, which in turn reduces the asynchrony between action and the sensory cue. The dominance of the tactile cue in this task suggests that task-type influences the weighting of sensory cues and in the context of sensorimotor synchronization where the task is inherently automatic and predicable, tactile information contributes to motor performance more than visual information.

In Experiment 2, we aimed to examine whether cross-modal stimuli presented separately during the TBW influenced temporal error of sensorimotor synchronization. Our results showed higher temporal error (mean asynchrony) in movement execution when cross-modal stimuli were presented inside the TBW than outside the window, thus supporting our hypothesis. As with Experiment 1, the results from Experiment 2 also fit within the assumptions of the linear phase correction model, MLE model, and Bayesian causal inference. Multisensory stimuli that are perceived and integrated (bound) within the TBW are assigned a weight by the CNS according to the reliability of the sensory cue, and then attributed to a common cause. Across trials, this attribution of a common cause of the multisensory stimuli may form the participants’ prior knowledge (“priors”) for future trials. This prior knowledge and the reliability weighting of sensory cues enables the CNS to phase shift the action of finger tapping towards the cue with the highest reliability. In the context of our study, when cross-modal stimuli were received within the TBW, mean asynchrony difference from baseline (temporal error) was higher than when one stimulus in the cross-modal pair was inside the TBW and the other stimulus was outside the TBW. Higher mean asynchrony (difference from baseline) here refers to longer intervals between stimulus and response that are due to the presence of both stimuli, and not only the influence of one sensory modality over the other. These results indicate that cross-modal stimuli received within the TBW influences the timing of movement execution such that the action is phase shifted away from the first stimulus: This is a form of time averaging, where the temporal proximity of the stimuli can affect the temporal estimation for an action. However, as we found that temporal error was lower for the tactile-visual (tactile attend/visual irrelevant) condition than for visual-tactile (visual attend/tactile irrelevant) in both the 80- and 400-ms conditions, the degree to which the action is phase shifted may depend on the weighting of the sensory cue, which is in agreement with a MLE model of cue integration. In our case, asynchrony (temporal error) was lower when the tactile cue was dominant (i.e., in the tactile-visual condition) for both 80- and 400-ms conditions.

A further aim for Experiment 1 and 2 was to examine the relationship between sensorimotor synchronization and the TBW. Results from both Experiments showed that individuals who integrate multisensory information over a longer period (i.e., have wider TBWs) are less precise at synchronizing their movements than those who integrate multisensory information over a shorter period. This finding indicates that the width of the TBW is related to anticipating the timing of sensory information. When the TBW is narrow (i.e., shorter in duration), the ability to anticipate the timing of events is more accurate. In contrast, when the window is wider, an individual may anticipate that the event they are timing their action with occurs later than it actually does, thereby delaying the execution of the action and making asynchrony more positive. Although the timing for anticipatory actions might be separable from time perception in specific conditions (Marinovic & Arnold, 2012), it is likely that anticipatory timing and other co-occurring motor processes, such as planning and preparation, are also affected by the width of the TBW.

Given the temporal nature of both tasks (simultaneity judgement task and the sensorimotor synchronization task), it is likely that the relationship between the binding window and movement execution is facilitated by temporal processing. In line with this idea, previous research shows that neural temporal processing is related to the perception of simultaneity (Roach et al., 2011), and that temporal perception plays a key role in sensory processing and motor coordination (Buonomano & Karmarkar, 2002). This interdependent relationship between the sensory, motor and temporal processes is important for considering a link between sensory processing difficulties and motor dysfunction in clinical populations, such as autism spectrum disorder (ASD). In the context of ASD, there is likely a connection between sensory and motor function that is underpinned by temporal processing. In support of this three-way relationship between sensory perception, motor function and temporal processing, individuals with ASD experience a variety of sensory difficulties associated with processing (Beker et al., 2017; Tavassoli et al., 2014), integration (Brandwein et al., 2012, 2015; Russo et al., 2010; Stevenson et al., 2014a, b), and binding (Brock et al., 2002; Foss-Feig et al., 2010; Greenfield et al., 2015; Zhou et al., 2018), as well as motor deficits (Bhat et al., 2011; Calhoun et al., 2011; Cascio et al., 2012; Fournier et al., 2010a, b; Rinehart et al., 2006). These motor deficits extend to sensorimotor synchronization, with ASD participants showing higher sensorimotor synchronization variability compared with non-ASD participants (Morimoto et al., 2018; Murat Baldwin et al., 2021). Further, it has been suggested that individuals with ASD may have deficits in temporal synchrony (Murat Baldwin et al., 2021), temporal processing, and temporal perception (Allman, 2011; Allman et al., 2011; Casassus et al., 2019; Stevenson et al., 2016), and experience difficulty in detecting temporal changes in sensory stimuli (Brodeur et al., 2014; Falter et al., 2012). As individuals with ASD often have differences in cerebellum function (D'Mello & Stoodley, 2015; Mosconi et al., 2015)—a crucial neural region involved in subsecond and suprasecond timing mechanisms and motor control (Bijsterbosch et al., 2011; Grondin, 2010; Rao et al., 2001)—this difficulty with temporal perception and processing may be related to activity in the cerebellum. These findings in ASD further support the idea that temporal processing may aid in facilitating the relationship between multisensory integration and movement execution.

Although it was not a main aim of Experiment 1, our results inform whether sensorimotor synchronization performance was influenced by sub- and suprasecond interstimulus intervals. Despite seeing a difference in sensorimotor synchronization variability between sub- and suprasecond timing intervals, we believe this difference is not due to changes in activation patterns in neural regions associated with sub- and suprasecond timing intervals. Instead, the difference in variability between sub- and supratiming intervals might be driven simply by the fact that longer intervals are likely to have higher variability than shorter intervals. If the pattern of results for sensorimotor synchronization had been different between the sub- and suprasecond intervals, we could have inferred that neural regions were differentially activated, but this was not the case.

In conclusion, our results show that individuals’ who take longer to integrate multisensory information are more variable, and have larger temporal errors, when synchronizing actions with external sensory cues. When there is a temporal delay between multisensory stimuli that are bound in the TBW, the timing of motor execution is affected such that longer offsets between the onset of multisensory cues interferes with the temporal precision of the action (i.e., finger tap). Further, we have shown that tactile cues are weighted more strongly than visual cues as they are more intrinsically linked to motor timing and the performance of the action in sensorimotor synchronization tasks. These results have implications for clinical populations, such as ASD, in which there may be a relationship between difficulties with multisensory integration and differences in motor abilities.

### Supplementary Information

Below is the link to the electronic supplementary material.

Supplementary file1 (DOCX 123 KB)

## Data Availability

The datasets generated and analyzed during the current study are available from the corresponding author on reasonable request.

## Code availability

The code used in this study is available via contact with the corresponding author.
