# Supplementary material for: Tactile cues are more intrinsically linked to motor timing than visual cues in visual-tactile sensorimotor synchronization
Source: Atten Percept Psychophys. 2024 Jan 23;86(3):1022–37. doi: 10.3758/s13414-023-02828-9 (PMC11062975; doi:10.3758/s13414-023-02828-9)
Supplement: Supplementary file 1 — Supplementary file1 (DOCX 123 KB) [file 13414_2023_2828_MOESM1_ESM.docx]

**Supplementary material**

**Results**

**Experiment 1 – Task 1**

*Mean asynchrony*

Mean asynchrony was averaged across trials per condition for each participant. Mean asynchrony was analysed using a LMM with time (600 ms, 1200 ms) and stimulus type (visual, tactile and visual-tactile) as fixed effects and participants as the random effect. Results from the LMM showed a main effect of time (*F*(1, 2) = 3.93, *p* = 0.049) but no effect of stimulus type (F(1, 2) = 0.13, p = 0.877), and no time * stimulus type interaction (*F*(1, 2) = 0.083, *p* = 0.919). As the interaction was not significant, we did not conduct post-hoc tests.


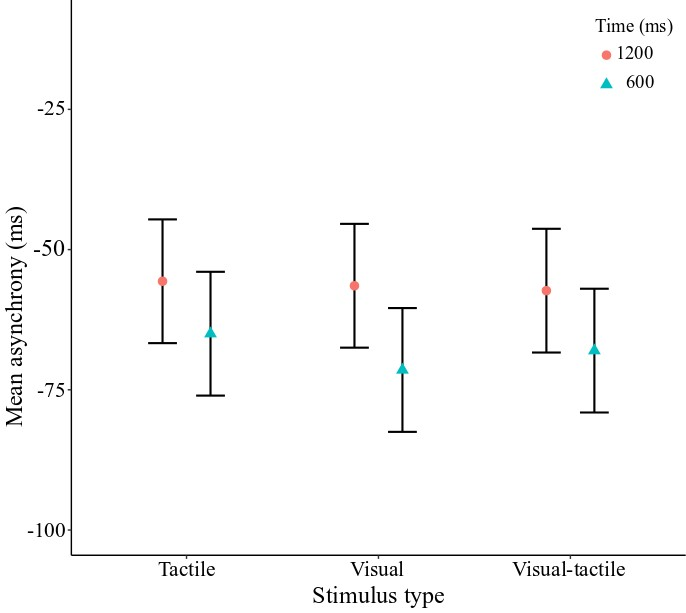


Figure 1: Sensorimotor synchronization mean asynchrony for tactile, visual and visual-tactile conditions for 600 and 1200 ms ISIs. Error bars represent the standard error of the model estimates.

**Experiment 2 – Task 1**

*Standard deviation asynchrony*

Sensorimotor synchronization variability was measured using the asynchrony standard deviation across trials for each condition. The standard deviation asynchrony was analysed using a LMM with time and condition as fixed effects and participants as the random effect. Results from the LMM showed a main effect of condition (*F*(1, 81) = 7.64, *p* = 0.007) but no effect of time (F(1, 81) = 2.40, p = 0.125), and no condition * time interaction (*F*(1, 81) = 3.66, *p* = 0.059) (see Figure 2). As the interaction did not reach significance, we did not conduct post-hoc testing.


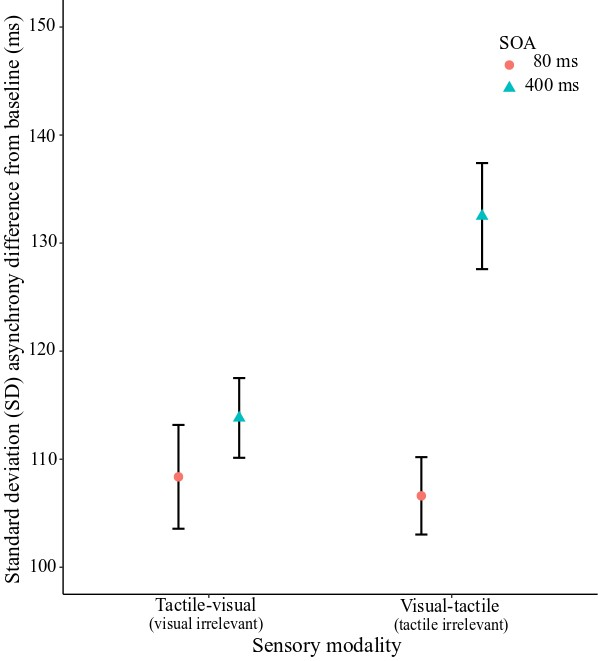


Figure 2: Sensorimotor synchronization asynchrony variability (standard deviation) difference score (cross-modal minus unimodal per condition) for tactile-visual (tactile attend/visual irrelevant) and visual-tactile (visual attend/tactile irrelevant) for SOA’s 80 ms and 400 ms. Error bars represent the standard error of the model estimates.
